# Geographically weighted regression analysis of incomplete basic childhood vaccination in Sub-Saharan Africa: Evidence from DHS, 2019–2024

**DOI:** 10.1371/journal.pone.0336498

**Published:** 2025-11-21

**Authors:** Tigist Kifle Tsegaw, Eyob Akalewold Alemu, Fetlework Gubena Arage, Zinabu Bekele Tadese, Eliyas Addisu Taye, Tsegasilassie Gebremariam Abate

**Affiliations:** 1 Department of Public Health Officer, Institute of Public Health, College of Medicine and Health Sciences, University of Gondar, Gondar, Ethiopia; 2 Department of Epidemiology and Biostatistics, Institute of Public Health, College of Medicine and Health Sciences, University of Gondar, Gondar, Ethiopia; 3 Department of Health Informatics, School of Public Health, College of Medicine and Health Sciences, Samara University, Semera, Ethiopia; 4 Department of Health Informatics, Institute of Public Health, College of Medicine and Health Sciences, University of Gondar, Gondar, Ethiopia; 5 Department of Maternal and Child Health, Lemi Kura Sub city Health Office, Addis Ababa City Administration Health Bureau, Addis Ababa, Ethiopia; Gabriele d'Annunzio University of Chieti and Pescara: Universita degli Studi Gabriele d'Annunzio Chieti Pescara, ITALY

## Abstract

**Background:**

Immunization is the safest way to protect against disease. Currently, vaccination saves more than 4 million lives annually. By 2030, 90% of people worldwide are expected to have received the basic immunizations, according to the Immunization Agenda 2030 (IA2030). However, in sub-Saharan Africa (SSA), only 54.1% of children receive the complete set of basic childhood vaccinations. Therefore, this study aims to assess the spatial variation of incomplete basic childhood vaccination and its determinants in SSA by using DHS data from 2019–2024.

**Method:**

For our study, we utilized a total of 28,045 weighted children from 16 selected SSA countries. The vaccination status was determined through both maternal recall and the use of vaccination cards. Spatial autocorrelations, hotspot analysis, spatial interpolation, and SaTScan analysis were conducted to explore the spatial distribution. Ordinary Least Squares (OLS) and Geographical Weighted Regression (GWR) were performed to identify the associated factors of partial immunization.

**Results:**

The pooled prevalence of partial immunization in SSA was 35.5% [95% CI: 28.49%, 42.51%]. Mauritania, Gabon, Côte d’Ivoire, central Tanzania, Liberia, and northeast Mozambique are among the hotspot regions that have been identified. Higher maternal education, female-headed households, maternal age between 15 and 24 years, absence of antenatal care, and urban residency were all found to be significant predictors with GWR analysis.

**Conclusion:**

In SSA, partial immunization shows a clustered spatial pattern with different hotspot areas. The use of maternal health services and socio-demographic factors has an impact on incomplete immunization. Improving vaccination coverage requires focused programs that address awareness creation and service utilization.

## 1. Introduction

Immunization is the most effective way to protect against disease. Measles, meningitis, pneumonia, tetanus, and polio are among the diseases that could cause severe illness and disability if we are not vaccinated. Children are especially vulnerable to infections due to their compromised immune systems, so it is crucial that they receive their vaccinations on time [1].

Immunization currently prevents over 4 million deaths each year. In the African region alone, vaccines save approximately 800,000 lives annually [1]. However, in 2023, 21 million children worldwide missed out on lifesaving diphtheria, pertussis, and tetanus vaccines, and 6.5 million were only partially vaccinated [2,3]. Additionally, one in five children in Africa did not receive basic, lifesaving immunizations like the DTP3 vaccine [4]. Basic childhood vaccines include BCG, pentavalent, polio, and measles [5].

In Africa, vaccine-preventable diseases (VPDs) such as measles, diphtheria, tetanus, polio, and pertussis—ailments that have been nearly eradicated in many high-income countries—affect over 30 million children under the age of five each year. These infections cause more than 500,000 deaths annually in Africa, accounting for approximately 58% of all global VPD-related child deaths [1,6]. An estimated 700,000 children under the age of five died from VPDs globally in 2018, with low- and middle-income countries accounting for nearly 99% of these deaths [7]. In 2023, VPDs were responsible for almost 30% of all under-five deaths in SSA, making the region’s under-five mortality rate nearly 14 times higher than that of Europe and 18 times higher than that of Australia and New Zealand [3,8].

Despite considerable efforts to increase immunization coverage across Africa over the past decade, routine immunization demand continues to face significant challenges. Most African countries are unlikely to meet their Sustainable Development Goals (SDG) as long as children remain at risk of outbreaks of vaccine-preventable diseases, which pose serious threats to their health and lives.[4]. Immunization Agenda 2030 (IA2030) aims to achieve 90% coverage and reduce the number of ‘zero-dose’ children globally to fewer than 6.5 million by 2030 [9]. However, in SSA, only 54.1% of children receive the complete set of basic childhood vaccinations, while 36.06% were partially immunized. Studies indicate that SSA continues to face significant challenges in meeting the IA2030 immunization targets [10–12]. Additionally, there is considerable variation in immunization coverage across different African countries [13,14].

The factors contributing to incomplete immunization include being a young mother, lacking knowledge about immunization, and having negative perceptions of vaccine side effects. Additionally, children of single mothers, those born without the presence of a skilled birth attendant, and those whose mothers did not receive postnatal care are more likely to experience incomplete immunization. Other contributing factors include poor maternal knowledge of routine immunization, living in rural districts, belonging to low-income families, and residing more than 30 minutes away from the nearest vaccination facility [15,16].

The spatial distribution of this condition and the factors contributing to it in SSA countries are not well addressed. Therefore, geographically weighted regression should be employed to investigate the spatial patterns and variation in immunization coverage among children aged 12–23 months in SSA countries. Identifying high-risk areas through an understanding of spatial distribution is essential to reducing the rate of incomplete vaccination. This approach will enable targeted interventions and more efficient resource allocation. Accordingly, this study aims to assess the spatial variation of incomplete basic childhood vaccination and its determinants in SSA using Demographic and Health Survey (DHS) data from 2019 to 2024.

## 2. Method and material

### 2.1. Study area and study period

**Fig 1:**
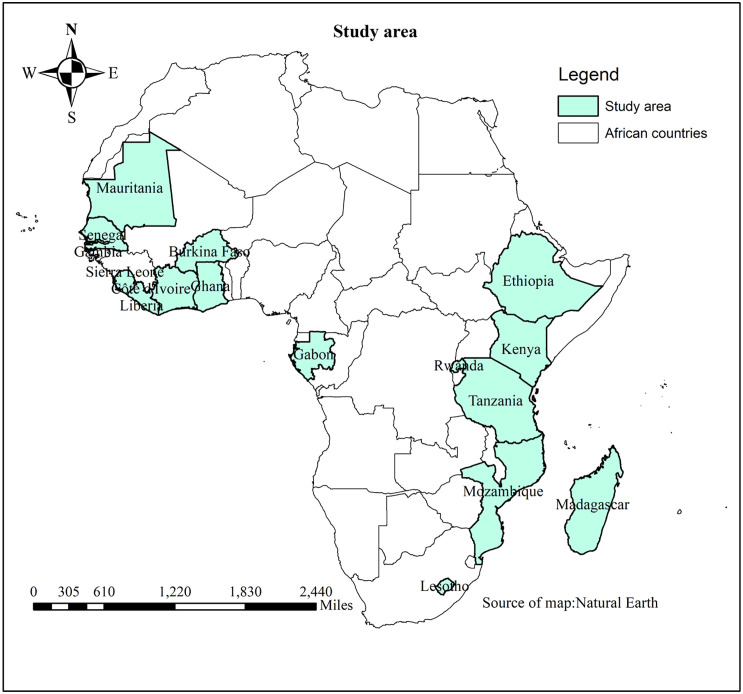
Study area.

The study was conducted in 16 Sub-Saharan African countries from 2019–2024 (Fig 1). The region comprises 47 countries with a total population of 1.07 billion. For this study, only countries with recent DHS data within the five-year period were included to ensure that the analysis reflects the most recent trends in child immunization across Sub-Saharan Africa. The countries included are Burkina Faso (2023), Côte d’Ivoire (2021), Ethiopia (2019), Gabon (2022), Gambia (2020), Ghana (2022), Kenya (2022), Lesotho (2024), Liberia (2019), Madagascar (2021), Mauritania (2021), Mozambique (2023), Rwanda (2020), Senegal (2023), Sierra Leone (2019), and Tanzania (2022). SSA region has the highest number of underdeveloped countries in the world and accounts for the greatest burden of deaths and disabilities due to infectious diseases.

### 2.2. Data source

The data for this study were obtained from the most recent DHS data conducted in Sub-Saharan African countries. The analysis included countries with available DHS data from the past five years (2019–2024).The dataset was downloaded in STATA format from the DHS website (http://www.dhsprogram.com). The DHS data are nationally representative and utilize four main standard model questionnaires (the Household Questionnaire, the Woman’s Questionnaire, and the Man’s Questionnaire) to collect data that are comparable across countries. These questionnaires cover basic health indicators such as marriage, fertility, mortality, family planning, reproductive health, child health, nutrition, and HIV/AIDS. The data includes records for men, women, kids, and household dataset records. For this study, we used Kids recode files.

### 2.3. Study design

This study utilized secondary data from the DHS, a nationally representative, community-based cross-sectional survey that collects information on population, health, and nutrition. These surveys are usually carried out roughly every five years and have sizable sample sizes, ranging from 5,000–30,000 households. Representativeness at the national, state or departmental, and residential (rural or urban) levels is assured by the sampling design.

### 2.4. Study population

The source population was all alive children aged 12–23 months in Sub-Saharan African countries, and the study population was all alive children aged 12–23 months in Sub-Saharan African countries during the study period.

### 2.5. Sample size determination and sampling procedure

The DHS program usually uses a two-stage stratified probability sampling design. Samples were typically stratified by geographic region and by urban/rural areas within each region. In the first stage, primary sampling units (clusters) were selected from the enumeration areas. In the second stage, a complete listing was conducted, and 25–30 households were selected from each cluster by using equal probability systematic sampling. Data are then collected from each selected household [17]. For our study, we use a total of 28,045 weighted children from 16 selected SSA countries (Table 1).

**Table 1:** Sample size from each selected Sub-Saharan African country.

Country name	Year	Unweighted sample	Weighted sample	Weighted percentage (%)
Burkina Faso	2021	2,300	2,285	8.15
Côte d’Ivoire	2021	1,907	1,802	6.43
Ethiopia	2019	989	1,009	3.6
Gabon	2019/21	1,247	1,257	4.48
Ghana	2022	1,969	1,819	6.49
Gambia	2019/20	1,580	1,452	5.18
Kenya	2022	3,672	3,316	11.83
Liberia	2019	1,059	931	3.32
Lesotho	2023/24	523	481	1.72
Madagascar	2021	2,333	2,321	8.28
Mauritania	2021	2,083	2,085	7.43
Mozambique	2022/23	1,673	1,730	6.17
Rwanda	2020	1,572	1,632	5.82
Sierra Leone	2018/19	1,848	1,817	6.48
Senegal	2023	2,040	1,931	6.89
Tanzania	2022	2,131	2,172	7.74
Total		28,926	28,045	

### 2.6. Variable of the study

#### 2.6.1. Outcome variable.

The dependent variable was incomplete immunization of basic childhood vaccination among children aged 12–23 months. Missing at least one among these basic childhood vaccines is considered as incomplete (partial) immunization. We recoded all basic vaccines as “0” and “1” for those who didn’t receive and those who received the recommended vaccine based on the mother’s report and immunization card, respectively. And then we added up all eight recoded variables, and then we recoded as “0” for those who didn’t receive any of the recommended basic vaccines, “1” for those who missed at least one of the basic vaccines, and “2” for fully vaccinated (for those who received all eight basic vaccines).

#### 2.6.2. Independent variables.

The independent variables for this study were maternal educational level, employment status, residence, household wealth, mothers’ media exposure, antenatal care (ANC) utilization, and place of delivery.

#### 2.6.3. Operational definition.

A fully vaccinated child is one who has received all the required doses of the basic childhood vaccines, including BCG, OPV1, OPV2, OPV3, Penta1, Penta2, Penta3, and MCV1. A child is considered incompletely immunized or partially immunized if they have missed at least one dose of these vaccines. Conversely, a non-vaccinated child is one who has not received any vaccines at all.

**Media exposure;** having exposure at least for one of the three (TV, radio and newspaper)

### 2.7. Data management and analysis

The data were kept, cleaned, recorded, appended, and analyzed using STATA version 14.2, while the spatial analysis was performed with ArcGIS 10.7.1. Descriptive statistics, including percentages, proportions, graphs, and frequency tables, are presented. Missing values were handled according to the DHS guidelines. The pooled prevalence of incomplete childhood vaccination was estimated using a random-effects model. The sample was weighted using the sampling weight variable v005 to make valid inferences. Multiple spatial analyses were undertaken to visualize and explore the geographical variation of incomplete immunization across regions from 2019 to 2024. The unit of analysis was a region (v024) of each country. The shapefile for regional boundaries was obtained from Natural Earth (http://www.naturalearthdata.com/). We calculated the weighted proportion of partial immunization for regions within each country using Microsoft Excel. The resulting regional data were then merged directly with the shapefile based on region identifiers to facilitate spatial analysis.

#### 2.7.1. Spatial Autocorrelation.

The global spatial autocorrelation (Global Moran’s I) was calculated to declare whether incomplete immunization uptake was dispersed, clustered, or randomly distributed in SSA. A spatial statistic called Global Moran’s I uses the complete dataset to generate a single output value between −1 and +1 in order to quantify spatial autocorrelation. A closer distance from −1 to Moran’s output suggests that the event of interest is dispersed, whereas a closer distance from +1 indicates clustering, and a closer distance from 0 suggests a random pattern. A statistically significant Moran’s I (p < 0.005) shows that the distribution of incomplete immunization uptake initiation is nonrandom (either clustered or dispersed).

#### 2.7.2. Hot Spot Analysis.

Hot Spot Analysis, or Getis-Ord Gi*, was utilized to identify areas exhibiting a concentrated occurrence of partial immunization. Regions displaying a pattern with a red color were interpreted as indicating a higher proportion of partial immunization, whereas a pattern with a blue color indicates a region with lower proportion of partial immunization.

#### 2.7.3. Spatial ordinary kriging interpolation.

Based on neighborhood measured data, the standard Kriging method of spatial interpolation was used to predict the percentage of incomplete vaccine uptake of unsampled sites. The Kriging method was preferred over other interpolation methods because an ideal interpolator providing a minimum mean error (ME) and root mean square error is kriging interpolation. An area shaded or interpolated in red was interpreted as a higher proportion of partial immunization.

#### 2.7.4 Spatial scan statistical analysis.

In the spatial scan statistical analysis, Bernoulli-based model was employed to identify statistically significant spatial clusters of partial immunization using SaTScan version 10.1 software.

Children with partial and no immunization were taken as cases, and those with full immunization were considered as controls to fit the Bernoulli model. The scanning window with maximum likelihood was the most likely performing (primary) cluster. For each identified cluster, the log-likelihood ratio (LLR) test statistic with its p-value, relative risk (RR), location radius, population, and cases were reported.

#### 2.7.5. Spatial regression analysis.

The Ordinary Least Squares (OLS) regression and Geographically Weighted Regression (GWR) statistical analysis were employed for exploring the spatial relationship between the partial immunization and explanatory variables.

2.7.5.1. Ordinary Least squares regression. The OLS global regression model operates under the assumption of uniform coefficients for each variable across the study area. It aims to determine the relationship between independent and dependent variables. Following an explanatory variable analysis, the OLS model was used to identify the most suitable predictor variables for the spatial variation in incomplete immunization uptake.

Before moving on to the local model, the six assumptions of the OLS model (the strength of R-square, the VIF value, the significance of each explanatory variable, the randomness of residuals, and the assurance of the statistical non-significance of Jarque-Bera statistics) were verified. Based on the Variance Inflation Factor (VIF) results, the multicollinearity was evaluated.

2.7.5.2. Geographically weighted regression. The GWR analysis was employed when the Koenker statistics became significant (p-value < 0.05), which means the relationships between the dependent and the independent variable change from location to location. In the GWR analysis, the coefficients of the explanatory variables take different values across the study area. Mapping the GWR coefficients associated with the explanatory variables, which are produced using the GWR, provides insight for targeted interventions.

### 2.8. Ethical consideration

This study was based on the existing survey data collected by the Demographic and Health Surveys (MEASURE DHS) project (www.measuredhs.com). The DHS program obtains ethical approval from the Institutional Review Board (IRB) of ICF International as well as from the relevant national ethics committee in each survey country. Informed written consent was obtained from all participants by DHS enumerators at the time of data collection an informed consent statement is read to the respondent, who may accept or decline to participate, and a parent or guardian must provide consent prior to participation by a child or adolescent. We obtained permission to access the raw DHS data upon request from the DHS Program (authorization letter number 184828), and the data were made available to us by the DHS data archivist in May 2023.

## 3. Results

### 3.1. Socio-demographic characteristics of the participants

A total of 28,045 weighted children aged 12–23 months were included in this study. Kenya contributed the largest proportion of the sample (11.8%), while Lesotho contributed the smallest (1.7%) (Table 1). Among the children, 52.2% were male, and 61.8% resided in rural areas. Approximately 36.1% of the mothers had no formal education, the majority of mothers (66.1%) were married, and 55.5% were employed (Table 2).

**Table 2:** Socio-demographic characteristics of the participants to assess the spatial distribution of partial immunization among children aged 12-23 month in SSA.

characteristics	Frequency	Percentage (%)
Sex of child		
Male	14,634	52.18
Female	13,411	47.92
Residence		
Urban	10,713	38.2
Rural	17,332	61.8
Employment		
Employed	15,003	55.49
Unemployed	12,032	44.51
Education		
No	10,444	36.11
Primary	9,138	31.59
Secondary	8,031	27.76
Higher	1,313	4.54
Mothers age		
15-24	9,184	32.75
25-35	14,002	49.93
> 35	4,858	17.32
Marital status		
Single	9,495	33.86
Married	18,549	66.14

### 3.2. Pooled prevalence of partial immunization among children aged 12–23 months in SSA

**Fig 2:**
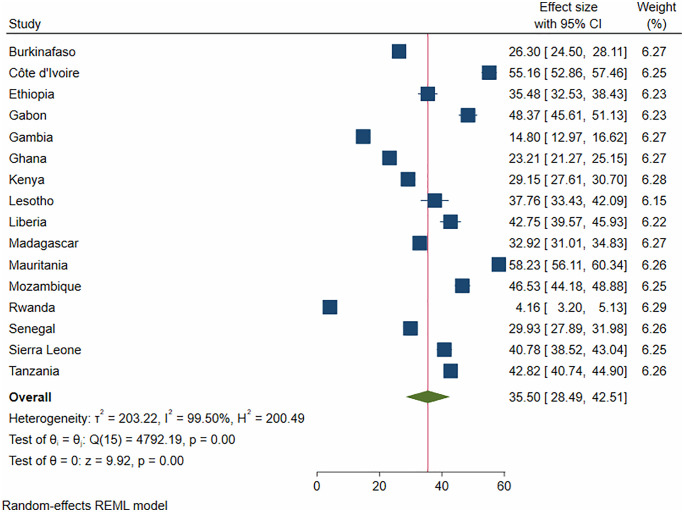
Forest plot for partial immunization among children aged 12-23 months in SSA.

The pooled prevalence of incomplete basic childhood vaccination among children aged 12–23 month in 16 SSA countries was 35.5% [95%CI; 28.49%, 42.51%] (Fig 2).

### 3.3. Each basic vaccine uptake

**Fig 3:**
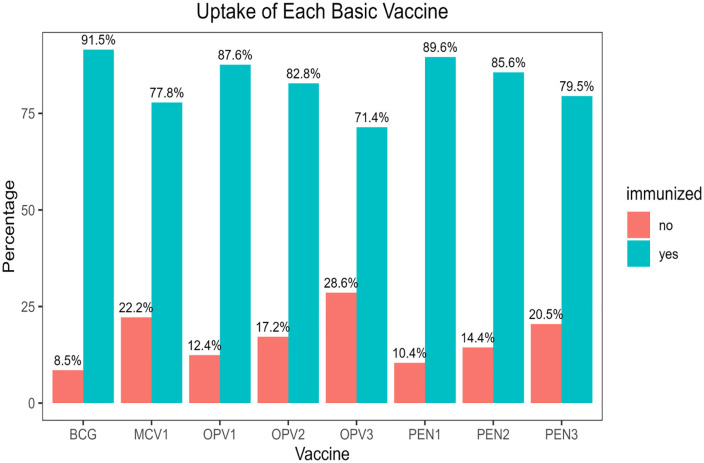
Basic childhood vaccine uptake among children aged 12-23 months in SSA from 2019-2024.

The BCG vaccine had the highest uptake, with 91.5%, followed by the pentavalent vaccine one (89.6%). The OPV3 vaccine had the lowest uptake, with 71.4%, suggesting that vaccines administered later in the schedule are more likely to be missed (Fig 3).

### 3.4. Partial immunizations along explanatory variables among children aged 12–23 months in SSA

Among children with partial immunization, 38.64% had mothers with no formal education, compared to 34.61% whose mothers had primary education, 32.42% with secondary education, and 25.84% with higher education. Children of unemployed mothers had a higher rate of partial immunization (38.0%) than those of employed mothers (32.18%).

Partial immunization rates also varied according to maternal antenatal care attendance and place of birth. Among children whose mothers had no ANC visits, 37.83% were partially immunized, compared to 34.83% for those with 1–4 visits and 34.15% for 5–20 visits. Children born at home had a higher rate of partial immunization (39.28%) than those born in health facilities (33.7%). Regarding media exposure, 32.1% of children whose mothers had media exposure were partially immunized, compared to 41.06% of children whose mothers had no media exposure. Differences by residence were slight, with 36.61% partially immunized in urban areas versus 33.68% in rural areas. Partial immunization was also higher among children from poor households (37.49%) compared to those from middle-income (34.63%) and wealthy households (33.8%) (Table 3).

**Table 3:** Incomplete basic childhood vaccination among children aged 12-23 months in SSA from 2019-2024.

Variable	Immunization uptake						Total
	No		partial		Full		
	Frequency	Percentage (%)	Frequency	Percentage (%)	Frequency	Percentage (%)	
**Age**							
15-24	687	7.48	3,384	36.85	5,113	55.68	9,184
25-35	824	5.89	4,744	33.88	8,434	60.23	14,002
> 35	270	5.55	1,629	33.55	2,960	60.9	4,859
**Education**							
No education	910	9.87	3,568	38.64	4,754	51.49	9,232
Primary	540	5.49	3,150	34.61	5,412	59.45	9,102
Secondary	280	3.47	2,617	32.42	5,178	64.11	8,077
Higher	49	2.99	422	25.84	1,162	71.17	1,634
**Employment**							
Unemployed	878	7.3	4,572	38.0	6,583	54.71	12,033
Employed	705	4.7	4,827	32.18	9,469	63	15,002
**ANC**							
No ANC	583	27.6	799	37.83	730	34.56	2,112
1-4	725	5.46	4,625	34.83	7,930	59.71	13,280
5-20	373	3.23	3,918	34.15	7,183	62.6	11,474
**Place of birth**							
Home	1,059	19.2	2,167	39.28	2,291	41.52	5,518
Health facilities	721	3.2	7,592	33.7	14,213	63.10	22,527
**Media**							
Yes	704	3.71	6,097	32.1	12,190	64.19	18,992
No	879	10.93	3,303	41.06	3,862	48.01	8,044
**Residence**							
Urban	478	4.47	3,922	36.61	6,313	58.93	10,713
Rural	1,302	7.51	5,837	33.68	10,193	58.81	17,332
**Wealth index**							
Poor	705	11	2,405	37.49	3,303	51.5	6,413
Middle	296	5.39	1,901	34.63	3,292	59.98	5,489
Rich	779	4.82	5,453	33.78	9,911	61.39	16,143

### 3.5. Spatial distribution of partial immunization among children aged 12–23 months in SSA from 2019–2024

The red color indicates regions with a higher prevalence of incomplete immunization. Although this pattern was observed across most of the area, it was particularly high in Mauritania, northern Kenya, central Tanzania, Gabon, Côte d’Ivoire, central Ethiopia, and northern Mozambique. The green spots indicate regions with a lower proportion of incomplete immunization, primarily found in Rwanda, Burkina Faso, Ghana, and Kenya (S1 Fig).

### 3.6. Spatial autocorrelation analysis of partial immunization among children aged 12–23 in SSA

**Fig 4:**
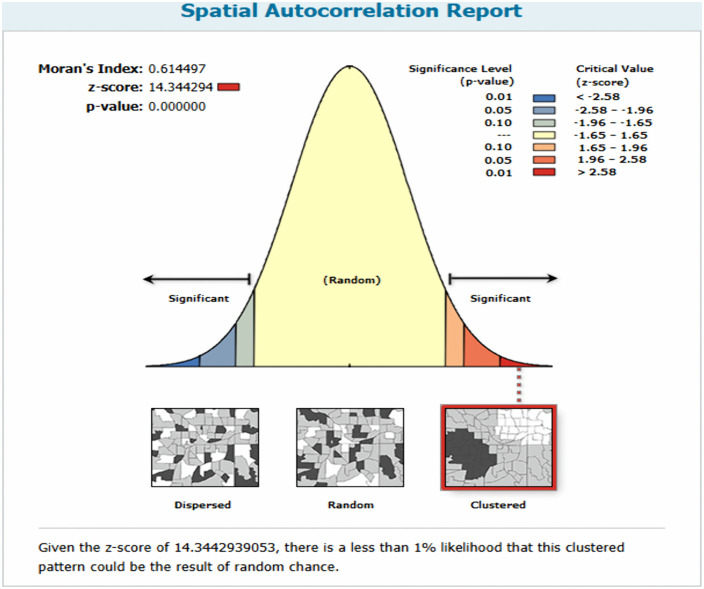
Spatial Autocorrelation of partial immunization among children aged 12-23 months in SSA from 2019-2024.

This study showed that the spatial pattern of partial immunization among children aged 12–23 in SSA was found to be clustered, with a Global Moran Index value of 0.61 (P-Value = 0), which implies that there is a less than 1% likelihood that this clustered pattern could be the result of random chance (Fig 4).

### 3.7. Hot spot analysis of partial immunization among children aged 12–23 months in SSA 2019–2024

**Fig 5:**
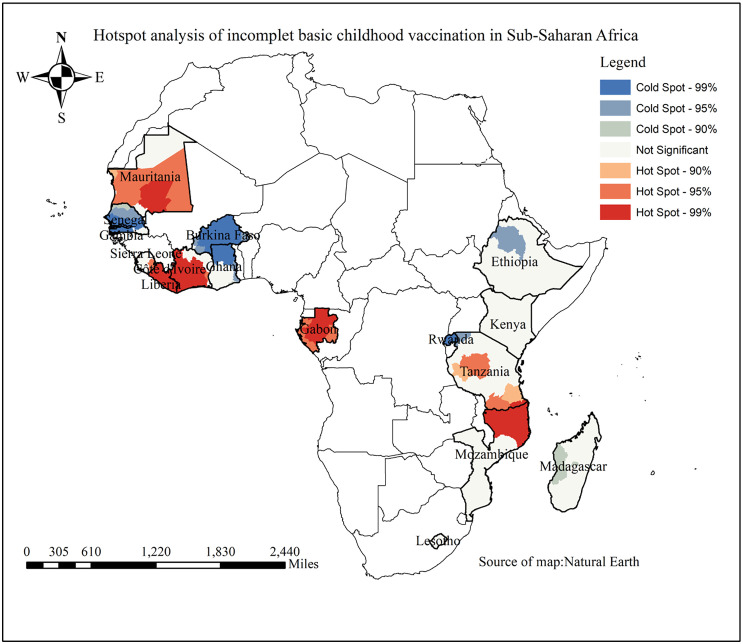
Hotspot analysis of partial immunization among children aged 12-23 month in SSA from 2019-2024.

Hot spot and coldspot analysis was conducted to identify areas with a high and low proportion of partial immunization among children aged 12–23 months in SSA. Areas highlighted in red represent regions with a significantly high proportion of partial immunization and are found in Mauritania, Gabon, Côte d’Ivoire, central Tanzania, Liberia, and northeastern Mozambique. In contrast, areas shown in blue color indicate a region with a low proportion of partial immunization, primarily observed in northern Ethiopia, northern Ghana, Senegal, Gambia, Rwanda, and Burkina Faso (Fig 5).

### 3.8. Spatial kriging interpolation of partial immunization among children aged 12–23 months in SSA

The map displays continuous surfaces interpolated using the Kriging method, which predicts unknown values of partial immunization at unsampled locations. The red color indicates a high likelihood of partial immunization, particularly in Mauritania, Côte d’Ivoire, Gabon, northern Mozambique, Liberia, and central Tanzania. In contrast, the green color indicates the lowest likelihood of partial immunization, mainly observed in Burkina Faso, Ghana, Madagascar, Senegal, and Ethiopia (S2 Fig).

### 3.9. Sat scan analysis of partial immunization among children aged 12–23 months in SSA from 2019–2024

**Fig 6:**
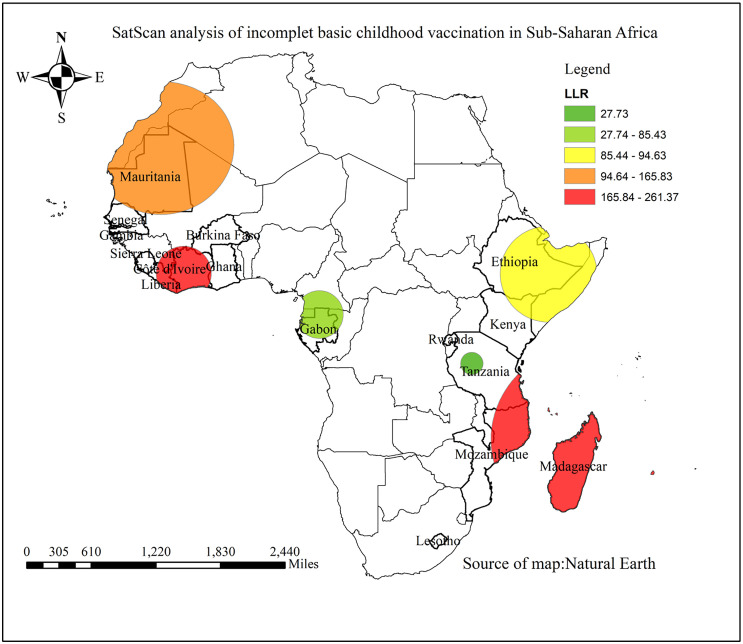
Sat Scan analysis of partial immunization among children aged 12-23 month in SSA from 2019-2024.

The spatial SAT scan analysis identified a total of seven significant clusters at a 0.05 significance level. The primary, or most likely, cluster was located northeast of Mozambique, Madagascar, and southeast Tanzania, centered at (15.319672° S, 48.305551° E), with a radius of 1,379.08 km, a log likelihood ratio of 261, and a relative risk of 1.53 (p < 0.001). This indicates that a child living within this spatial window has a 1.53 times higher risk of being unvaccinated or partially vaccinated compared to those outside the window. The secondary cluster was located in Liberia and Côte d’Ivoire, centered at (7.018640° N, 6.597574° W), with a radius of 416.34 km, a log likelihood ratio of 227, and a relative risk of 1.44 (p < 0.001). This suggests that a child residing within this window has a 1.44 times higher risk of being non- or partially immunized than those outside the window (Fig 6).

### 3.10. Ordinary least square regression analysis

The explanatory regression analysis identified five independent variables: higher maternal education, female-headed households, maternal age between 15 and 24 years, absence of antenatal care, and urban residency. This selection resulted in a higher adjusted R² of 28%. These variables were subsequently entered into OLS regression analysis. The OLS diagnostics for multicollinearity among the independent variables revealed a variance inflation factor (VIF) value of less than 10.

In the OLS analysis, the model explained 27.57% of the variation in partial immunization among children aged 12–23 months in SSA, with an AIC value of −315.26. Both the joint F-statistic and the p-value for the Wald statistics were less than 0.001, indicating that the model is statistically significant. The spatial distribution of the residuals was not normally distributed, which was statistically significant (p < 0.000). The Koenker statistics were statistically significant, indicating that the relationship between the independent and dependent variables was non-stationary or heterogeneous across the study area. This finding justifies the need to conduct GWR. From the OLS model, higher maternal education, urban residence, maternal age between 15 and 24, lack of ANC use, and female-headed household were statistically significant with partial immunization among children aged 12–23 months in SSA (Table 4).

**Table 4:** The ordinary least regression analysis results.

Variable	Coefficient	Robust SE	Robust t statistic	Robust p value	VIF
Intercept	0.108	0.037	2.89	0.000	
Higher education	−0.949	0.316	−2.99	0.003	1.32
Urban	0.185	0.039	4.7	0.000	1.28
15-24	0.405	0.101	7.18	0.000	1.71
No ANC	0.347	0.109	4.00	0.001	1.26
Female household headship	0.228	0.070	3.22	0.001	1.48
Ordinary Least Regression Diagnostic Analysis					
Number of observation	237		Adjusted R-squared		0.2756
Joint F-statistics	18.97		Prob (>F), (5, 231)df		0.000
Joint Wald statistics	92.51		Prob (>chi-squared).(5)df		0.000
Koenker(BP)statistics	20.26		Prob (>chi-squared).(5)df		0.000
Jarque-Bera	1.45		Prob (>chi-squared).(5)df		0.000

### 3.11. Geographically weighted regression analysis

The GWR analysis demonstrated a significant improvement over the OLS model. The AIC value decreased from AIC = −315.26 to −383.23, a difference of 67.84, indicating that the GWR better explains the spatial heterogeneity of partial immunization among children aged 12–23 months. Additionally, the adjusted R² increased to 56.81%, showing that the GWR model improved the explanatory power of the OLS model by approximately 29.25% (Table 5).

**Table 5:** Model comparison between OLS and GWR model.

Model comparison parameters	OLS model	GWR model
AIC	−315.26	−383.23
Adjusted R-squared	0.2757	0.5681

**Fig 7:**
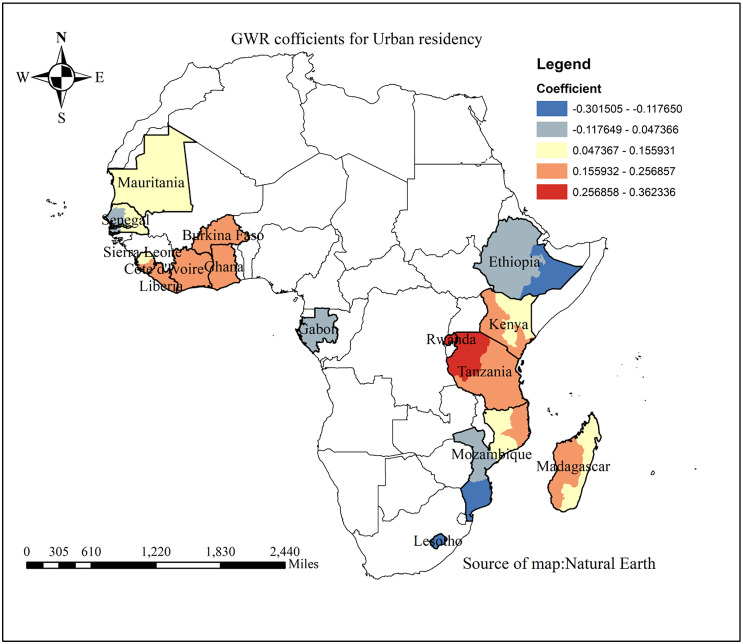
GWR coefficient for the association between partial immunization and place of residence in SSA from 2019-2024.

Urban residence was inversely associated with the proportion of partial immunization among children aged 12–23 months in eastern Ethiopia, and Mozambique, as indicated by the blue shading. In contrast, the red shading denotes a positive association between partial immunization and urban residence, primarily observed in Tanzania, Burkina Faso, Rwanda, Ghana, Côte d’Ivoire, and Liberia (Fig 7).

**Fig 8:**
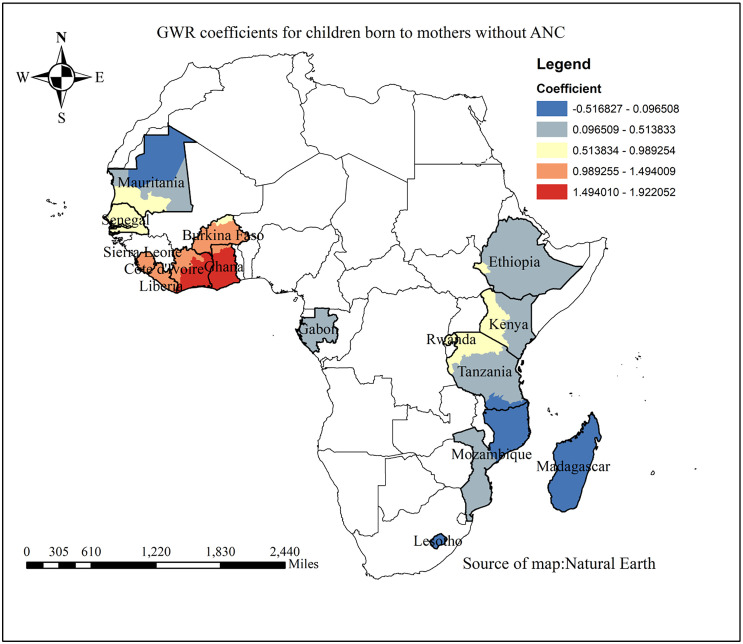
GWR coefficient for the association between partial immunization and non-ANC utilization in SSA from 2019-2024.

The probability of children receiving incomplete vaccinations increased with proportion of pregnant women who did not receive ANC. The map’s gray, yellow, and red shading indicates a gradient from weaker to stronger associations, illustrating this relationship. The countries of Ghana, Burkina Faso, Côte d’Ivoire, and Liberia exhibited the strongest correlations, highlighted in red. In contrast, as shown by the blue shading, no significant association was found in Madagascar, Mozambique, and Mauritania (Fig 8).

**Fig 9:**
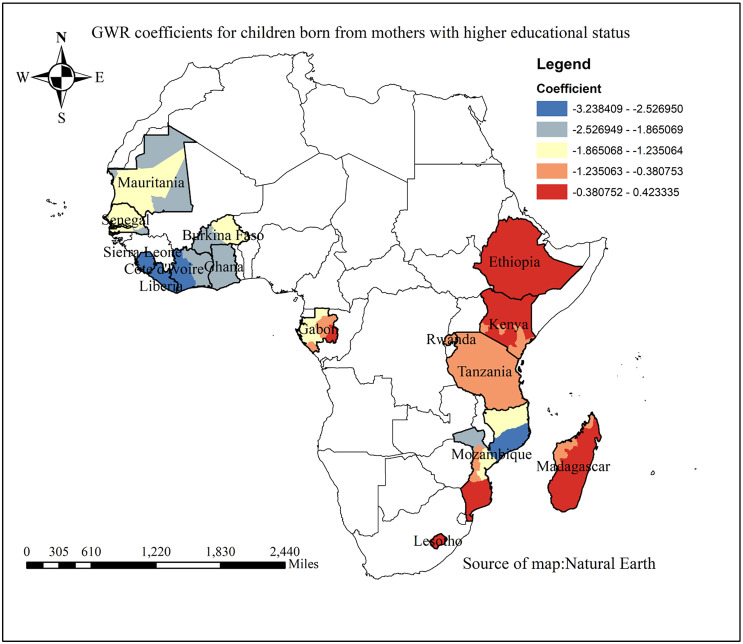
GWR coefficient for the association between partial immunization and higher maternal education in SSA from 2019-2024.

The map illustrates a correlation between higher maternal educational status and children’s incomplete vaccinations. The blue-shaded areas indicate the strongest negative associations, where children of mothers with higher education levels are less likely to have incomplete vaccinations. These areas include eastern Mozambique, Côte d’Ivoire, and Liberia. Conversely, weaker associations are observed in Tanzania, eastern Gabon, and western Madagascar. In regions shaded orange or light red, the protective effect of maternal education is less pronounced. Red-shaded areas with no statistically significant association indicate that maternal education has no statistically significant effect on incomplete immunization, as seen in Kenya, Ethiopia, Madagascar, and southern Mozambique (Fig 9).

As indicated by the blue shading, the risk of children in Gabon and southern Madagascar being partially immunized decreased as the percentage of mothers aged 15–24 increased. In contrast, Tanzania, Mozambique, Kenya, Burkina Faso, Ethiopia, northern Madagascar, Sierra Leone, Côte d’Ivoire, Ghana, and Liberia showed a positive correlation with partial immunization. The map illustrates a gradient of association strength, ranging from weak to strong, using light blue, yellow, orange, and red shading (S3 Fig).

The sex of the household head was another variable considered. The blue shading on the map indicates that in Gabon and eastern Ethiopia, being the head of a household was a protective factor against partial immunization. Conversely, the yellow, orange, and red shading in central and southern Tanzania, northern Mozambique, Mauritania, Senegal, Sierra Leone, Côte d’Ivoire, Burkina Faso, and Ghana indicates a positive association with partial immunization (S4 Fig).

## 4. Discussion

This study assessed the spatial variation and its determinants in SSA. Region’s immunization services encountered major obstacles during the COVID-19 pandemic. The COVID-19 pandemic caused a historic decline in childhood vaccination rates, with a 5% drop in DTP3 coverage from 2019 to 2021. In 2021, 25 million children missed DTP doses, including 12.7 million in Africa, highlighting the vulnerability of sub-Saharan Africa’s health system and significant setbacks in global vaccination efforts [18]. Recent reductions in USAID funding for Global Alliance for Vaccines and Immunization (GAVI) and WHO have exacerbated existing vaccination issues particularly for low-income countries. This condition is projected to leave 75 million children at risk for VPDs, leading to over 1.2 million deaths in the next five years. Insufficient financial resources will result in fewer new vaccines, increasing the likelihood of outbreaks. Accurate vaccine coverage estimates are essential for effective response; however, the suspension of DHS by USAID hinders monitoring and evidence-based studies, threatening years of progress against VPDs and potentially reducing future vaccination coverage [19,20]. Thus, it may be imperative to adopt alternative strategies, such as strengthening routine health information systems, utilizing administrative and facility-level data, and collaborating with international organizations and donors to provide the necessary support.

According to this study, the overall pooled prevalence of incomplete basic childhood vaccination among children aged 12–23 months in 16 Sub-Saharan Africa countries was 35.5% [95%CI; 28.49%, 42.51%],meaning that approximately four out of every ten children in this age group did not receive the full set of WHO-recommended basic childhood vaccines. It varied significantly between countries, ranging from 4.21% in Rwanda to 56.3% in Mauritania. These findings surpass the WHO’s recommended threshold of 10% for vaccine incompletion rates and also higher than estimates reported in studies from Australia (20%) and Myanmar (25.8%) [21–23]. The reasons may include knowledge gaps and inaccuracies among various stakeholders, lack of trust, and difficulties in accessing reliable immunization services [24]. Building vaccine confidence through community-led initiatives is crucial to increasing coverage, especially those that identify and involve respected leaders. These relationships are crucial for establishing trust and bolstering vaccine uptake in communities [25]. Furthermore, with numerous healths, social, and financial advantages, consistent funding for immunization programs continues to be one of the most economical public health initiatives.

Meanwhile It was lower than study from Pakistan (46%) [26], Nigeria (69.6%) [27], and Indonesia (58.9%) [28]. This could be attributed to differences in study periods and the types of vaccines considered. However it was in line with study from India (32%) [29], previous study in Africa (35.5%), and Sub-Saharan Africa (35.1%) [30,31] where limited access to high-quality healthcare services, poor infrastructure, long distances to healthcare facilities, and shortages of healthcare workers may explain the similarities [32,33].

The spatial distribution of incomplete basic childhood vaccination among children aged 12–23 months displayed a clustered pattern. The identified hotspot area were Mauritania, Gabon, Lesotho, Côte d’Ivoire, Central Tanzania, Liberia, northeast of Mozambique, and northern part of Kenya, and Mozambique. This pattern may be attributed to variations in socio-demographic characteristics, differences in health system program implementation, and the attitudes and practices surrounding immunization across different countries [34].

From the GWR analysis, an inverse association was observed between urban residency and incomplete basic childhood vaccination in eastern Ethiopia, Gabon, and Mozambique. This may be because urban areas typically have better healthcare facilities for vaccinating children. However, a positive association was observed in Tanzania, Burkina Faso, Rwanda, Ghana, Côte d’Ivoire, and Liberia. This discrepancy may be explained by the heterogeneity within urban areas, while capital cities and well-served neighborhoods tend to have high coverage, poorer slums and peri-urban areas often face barriers such as limited healthcare access, overcrowding, and social inequalities. Consequently, urban residency does not uniformly guarantee higher vaccination coverage, and local context, including intra-urban disparities [35].

Our study found a positive association between not attending ANC follow-up and incomplete childhood immunization in Ghana, Burkina Faso, Côte d’Ivoire, and Liberia. The influence of maternal health service utilization, including ANC, on childhood immunization has also been reported in other studies [36–38]. This may be because not attending ANC reduces mothers’ contact with healthcare providers, which limits their access to important information on the benefits and schedules of childhood vaccination, thereby hindering timely and complete immunization.

The prevalence of partial immunization among children aged 12–23 months had a negative positive association with higher maternal educational status. As the proportion of mothers with higher education increased, the prevalence of partial immunization decreased in Eastern Mozambique, Côte d’Ivoire, Liberia, Eastern Gabon, Western Madagascar, and Tanzania. It is consistent with the study from Bangladesh, Togo, and Mali [39–41]. This could be due to maternal education enhances awareness of child health issues and improves mothers’ ability to access and utilize healthcare services, thereby increasing the likelihood that children receive full immunization and achieve better health outcomes [42].

The prevalence of partial immunization among children aged 12–23 months had a positive association with maternal age. As the proportion of mothers between the ages of 15–24 increased the prevalence of partial immunization increased in Tanzania, Mozambique, Kenya, Burkina Faso, Ethiopia, northern Madagascar, Sierra Leone, Côte d’Ivoire, Ghana, and Liberia. This finding was consistent with study conducted in SSA, East Africa and Ethiopia [10,43,44] which is associated with early child marriage, and poor health-seeking behavior of a mother with younger age might be the possible reasons.

Children from female household head had a positive association with partial immunization in central and southern Tanzania, northern Mozambique, Mauritania, Senegal, Sierra Leone, Côte d’Ivoire, Burkina Faso, and Ghana. Inequality was observed in immunization status due to female headship in previous study done in Ethiopia [45] and Bangladesh [46]. This could be due to the fact that females may be burdened with economic constraints, time pressures, and the responsibilities of managing the household, which can limit their ability to ensure their child receives complete vaccinations. While it had a negative association in Gabon and eastern Ethiopia, highlights the context-specific impact of these factors on partial immunization. The results of this study can be generalized to the general population as it used a large, nationally representative dataset. Furthermore, the examination of variable effects across various geographic locations was made by the application of GWR is the strength of this study. The study does, however, have a few limitations. Important factors that were not consistently available in every country, like employment, media exposure, and distance to medical facilities were not included.

## 5. Conclusion

Incomplete basic childhood vaccination remains a major public health concern in SSA, far exceeding WHO-recommended thresholds. The findings reveal a clustered spatial pattern of incomplete immunization, with distinct hotspots and cold spots across countries. Key determinants—such as female household headship, younger maternal age (15–24 years), lack of ANC follow-up during pregnancy, and urban residency—contribute significantly to the observed disparities. To improve vaccination coverage, context-specific interventions are needed. Strengthening maternal health service utilization, particularly ANC attendance, addressing intra-urban inequalities, and expanding community-based education and awareness initiatives through health workers could help close the immunization gap. Furthermore, regional collaboration and evidence-based policymaking should be prioritized to ensure that every child, regardless of location or background, receives complete and timely vaccination.

## Supporting information

S1 Fig Spatial distribution of partial immunization in SSA. (TIF)

S2 Fig Kiriging interpolation of partial immunization in SSA. (TIF)

S3 Fig GWR coefficient for the association between partial immunization and maternal age between 15–24. (TIF)

S4 Fig GWR coefficient for the association between partial immunization and female household headship. (TIF)
