# Supplementary figures and images for: Geographically weighted regression analysis of incomplete basic childhood vaccination in Sub-Saharan Africa: Evidence from DHS, 2019–2024
Source: PLoS One. 2025 Nov 21;20(11):e0336498. doi: 10.1371/journal.pone.0336498 (PMC12637957; doi:10.1371/journal.pone.0336498)

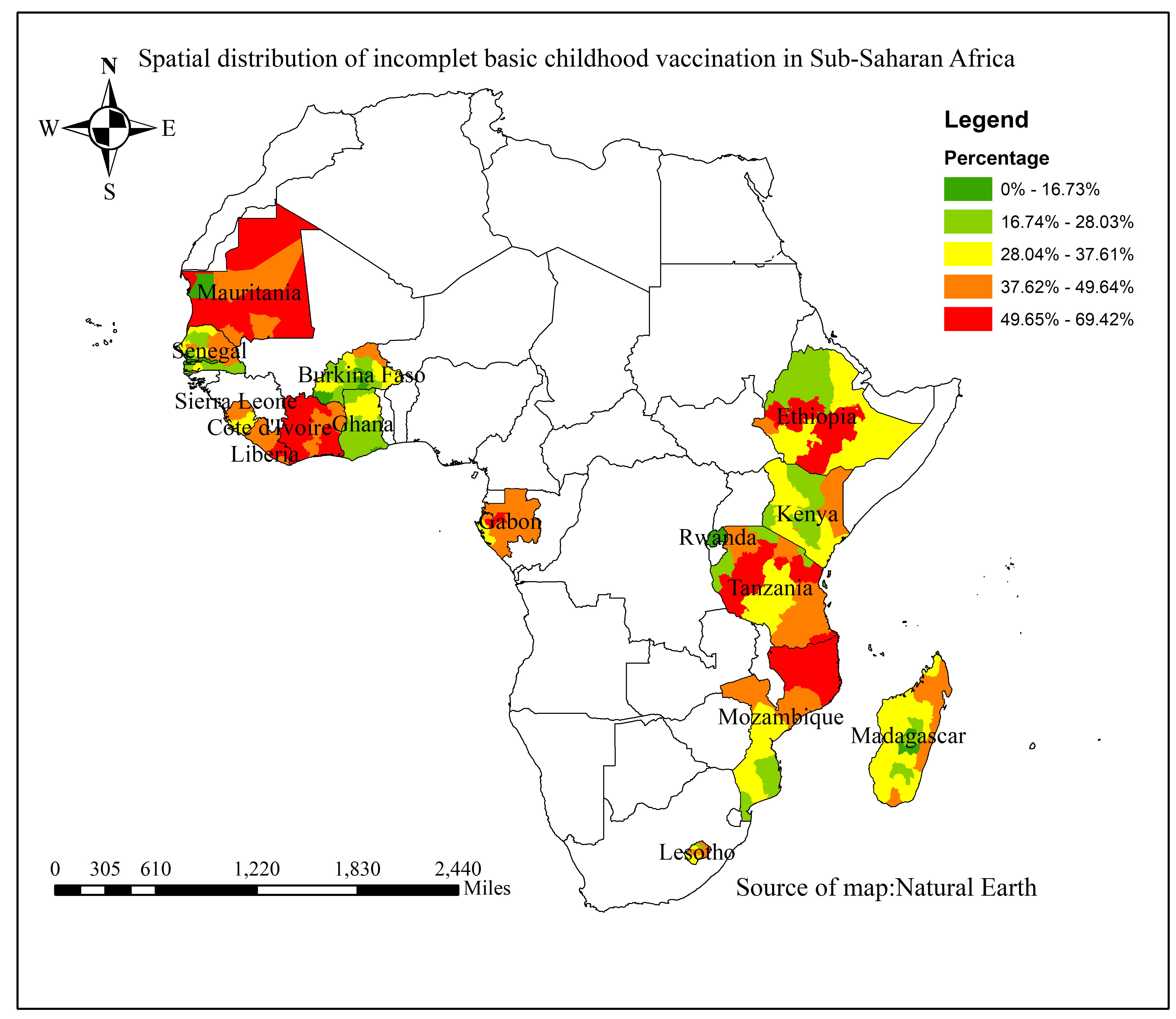

Supplement: S1 Fig — (TIF) [file pone.0336498.s001.tif]

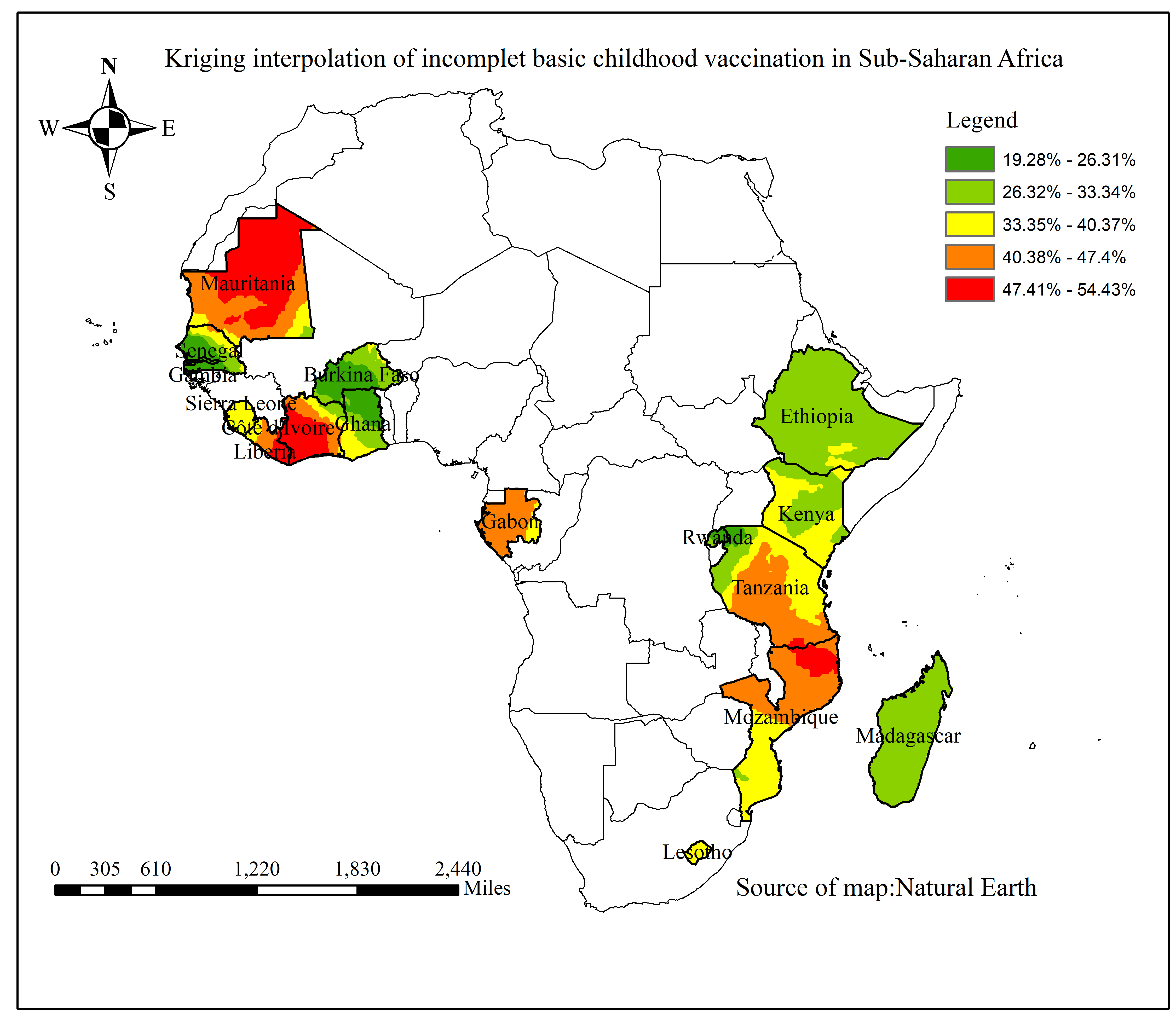

Supplement: S2 Fig — (TIF) [file pone.0336498.s002.tif]

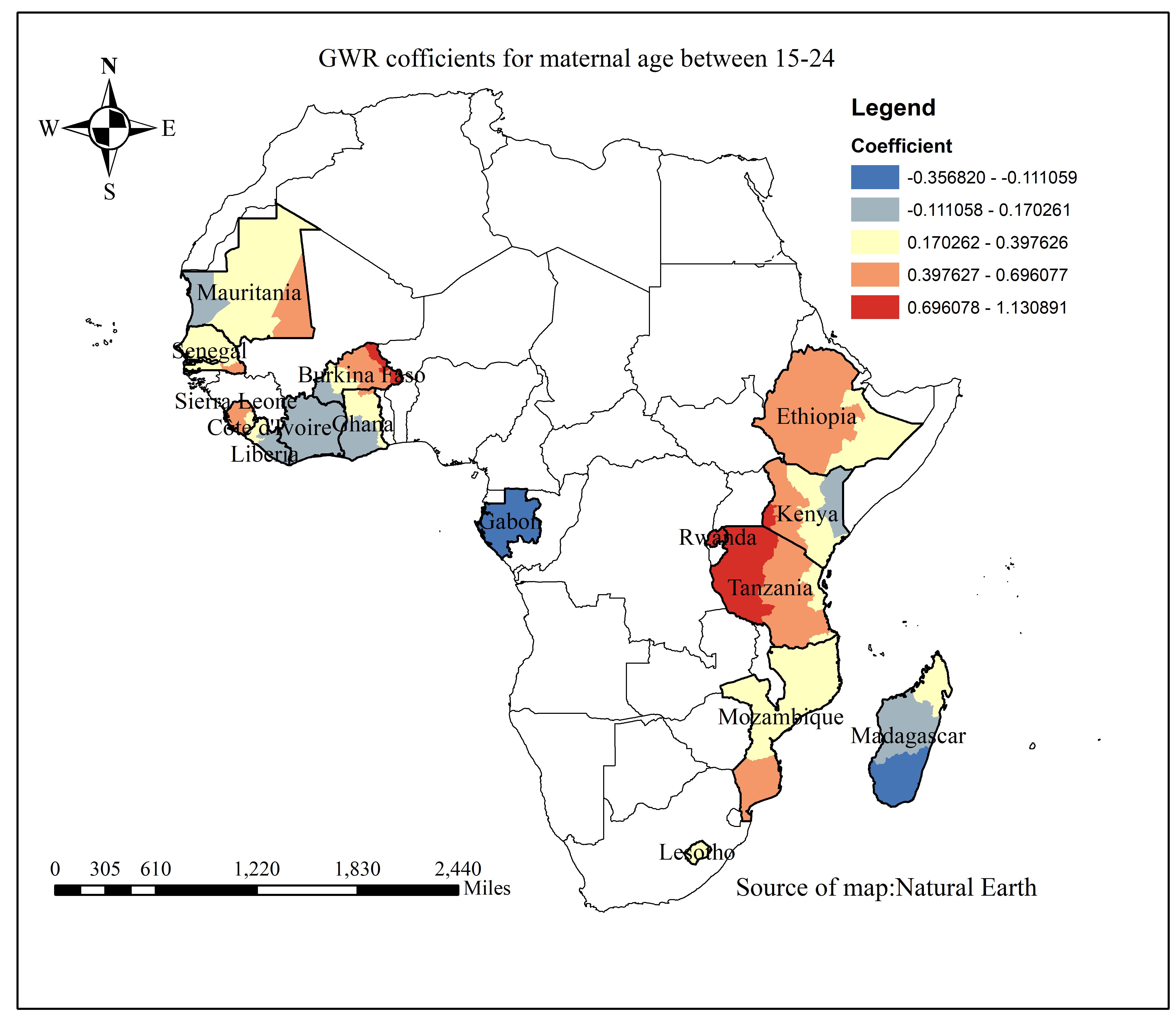

Supplement: S3 Fig — (TIF) [file pone.0336498.s003.tif]

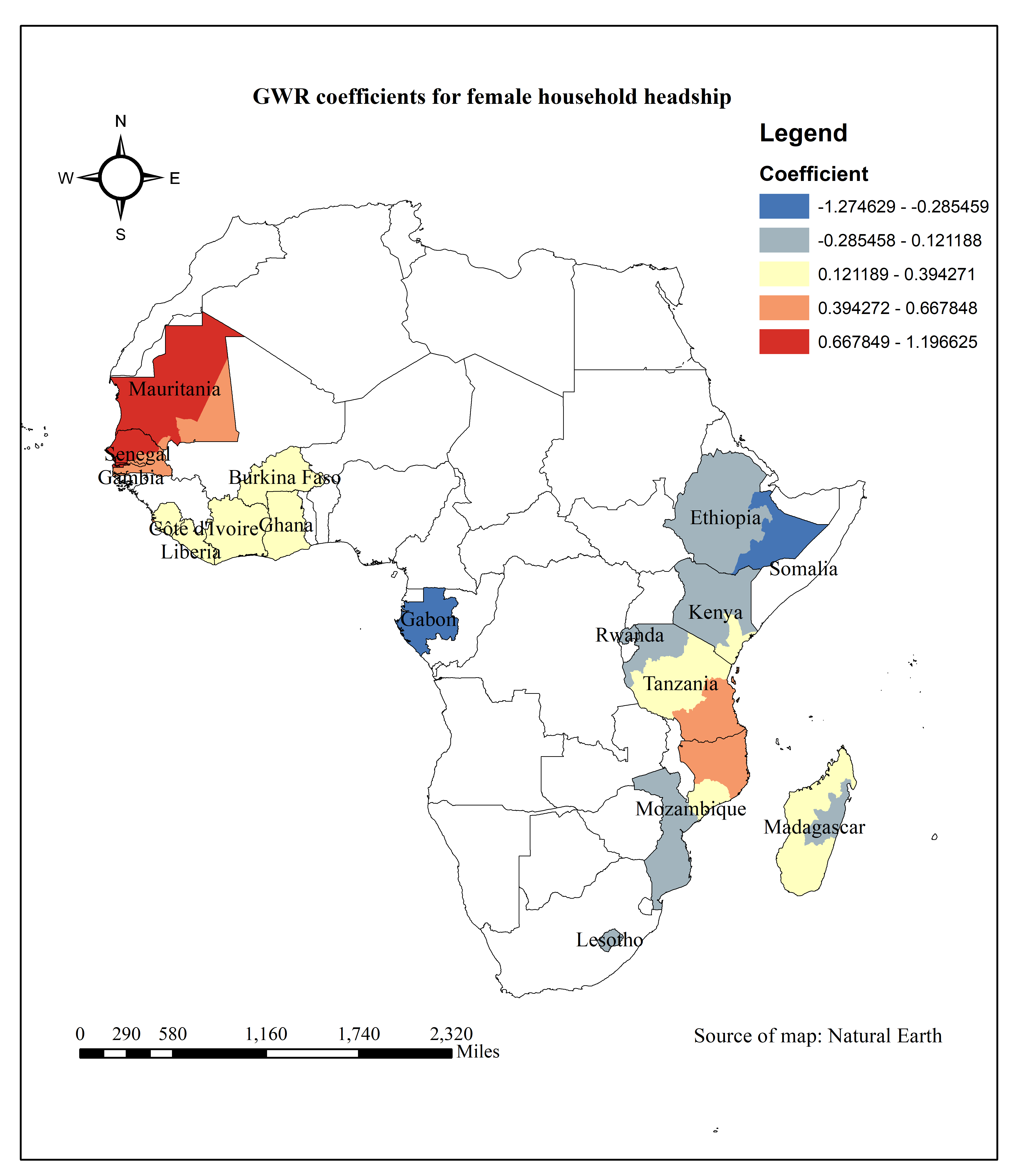

Supplement: S4 Fig — (TIF) [file pone.0336498.s004.tif]
